# Protective effects of intratracheally administered quercetin on lipopolysaccharide-induced acute lung injury

**DOI:** 10.1186/s12931-014-0150-x

**Published:** 2014-11-21

**Authors:** Koji Takashima, Miyoko Matsushima, Katsunori Hashimoto, Haruka Nose, Mitsuo Sato, Naozumi Hashimoto, Yoshinori Hasegawa, Tsutomu Kawabe

**Affiliations:** Department of Respiratory Medicine, Nagoya University Graduate School of Medicine, Nagoya, Japan; Department of Pathophysiological Laboratory Sciences, Nagoya University Graduate School of Medicine, 1-1-20 Daikou-minami, Higashi-ku, Nagoya 461-8673 Japan

**Keywords:** Quercetin, Heme oxygenase 1, Intratracheal administration, Alveolar macrophage, Acute lung injury

## Abstract

**Background:**

Acute respiratory distress syndrome (ARDS) can result in a life-threatening form of respiratory failure, and established, effective pharmacotherapies are therefore urgently required. Quercetin is one of the most common flavonoids found in fruits and vegetables, and has potent anti-inflammatory and anti-oxidant activities. Quercetin has been demonstrated to exhibit cytoprotective effects through the induction of heme oxygenase (HO)-1. Here, we investigated whether the intratracheal administration of quercetin could suppress lipopolysaccharide (LPS)-induced acute lung injury (ALI) in mice as well as the involvement of HO-1 in quercetin’s suppressive effects.

**Methods:**

Mouse model of ALI were established by challenging intratracheally LPS. The wet lung-to-body weight ratio, matrix metalloproteinase (MMP)-9 activities, and pro-inflammatory cytokine productions, including tumor necrosis factor (TNF)-α, interleukin (IL)-1β, and IL-6 in bronchoalveolar lavage fluid (BALF) were examined in ALI mice with or without quercetin pretreatment. We also examined the effects of quercetin on LPS stimulation in the mouse alveolar macrophage cell line, AMJ2-C11 cells.

**Results:**

Intratracheal administration of quercetin decreased the wet lung-to-body weight ratio. Moreover, quercetin decreased MMP-9 activity and the production of pro-inflammatory cytokines in BALF cells activated by LPS in advance. We determined the expression of quercetin-induced HO-1 in mouse lung, e.g., alveolar macrophages (AMs), alveolar and bronchial epithelial cells. When AMJ2-C11 cells were cultured with quercetin, a marked suppression of LPS-induced pro-inflammatory cytokine production was observed. The cytoprotective effects were attenuated by the addition of the HO-1 inhibitor SnPP. These results indicated that quercetin suppressed LPS-induced lung inflammation, and that an HO-1-dependent pathway mediated these cytoprotective effects.

**Conclusions:**

Our findings indicated that quercetin suppressed LPS-induced lung inflammation, and that an HO-1-dependent pathway mediated these cytoprotective effects. Intratracheal administration of quercetin will lead to new supportive strategies for cytoprotection in these serious lung conditions.

## Introduction

Acute respiratory distress syndrome (ARDS) continues to be major causes of mortality in the intensive care units, even though mechanical ventilation with low tidal volume [1], early neuromuscular blockade [2], and prone positioning [3] have been shown to reduce mortality from ARDS. No pharmacotherapy has proven effective in decreasing mortality in adult patients with ARDS [4]. The pathophysiology of ARDS involves inflammation with diffuse alveolar damage, formation of hyaline membranes, increased capillary permeability, interstitial edema, and influx of circulating inflammatory cells [5]. Although neutrophil influx and activation within the lung are important factors in the pathogenesis of ARDS, alveolar macrophages (AMs) and alveolar and bronchial epithelial cells are also involved in the disease process [5,6]. In particular, increasing evidence demonstrates that AMs contribute to the modulation of inflammatory responses and the resultant lung injury [7,8]. Pro-inflammatory cytokines, including tumor necrosis factor (TNF)-α, interleukin (IL)-1β, and IL-6, secreted by AMs stimulate neutrophils, and these activated neutrophils release oxidants, proteases, leukotrienes, and platelet activating factors, resulting in the development of ARDS [5].

Lipopolysaccharide (LPS), a component of the cell wall of Gram-negative bacteria, can induce inflammatory responses and disturb immune system function [9]. Intratracheal administration of LPS has gained wide acceptance as a clinically relevant model of ARDS in mice [10].

Quercetin is one of the most abundant dietary flavonoids and is found in a broad range of fruits, vegetables, and beverages. Quercetin has been demonstrated to have potent anti-inflammatory and anti-oxidant activities [11,12]. We reported that quercetin exhibited cytoprotective effects through the induction of heme oxygenase (HO)-1 [11,13,14]. HO-1 is a stress-inducible protein and catalyzes the rate-limiting step in the degradation of heme to biliverdin, carbon monoxide (CO), and ferrous iron [15].

ARDS often develops in intubated patients. During intubation management, the antioxidants supplied from a regular diet cannot be taken in. Therefore, we first focused on the prophylactic effects of quercetin to prevent the development of ARDS in high-risk patients. To qualify as a prophylactic treatment, it must be harmless, relatively inexpensive, and easily and widely applicable. It is thought that quercetin fulfills these conditions. Moreover, we considered that local administration to the lung could more effectively reveal the effects of quercetin, which is not easily absorbed, in the lung.

In this study, we investigated whether the intratracheal administration of quercetin could suppress LPS-induced acute lung injury (ALI).

We also investigated the involvement of HO-1 in the suppressive effects of quercetin.

## Materials and methods

### Reagents

LPS from *Klebsiella pneumoniae* LEN-1 (O3:K1^−^) was kindly donated by Prof. T. Hasegawa (Aichi Medical University School of Medicine, Aichi, Japan). Quercetin was obtained from Sigma (St. Louis, MO). Tin protoporphyrin IX (SnPP) was obtained from Frontier Scientific (Carnforth, UK).

### Cell culture

The mouse AM cell line, AMJ2-C11, and the mouse alveolar epithelial cell line, LA-4, were purchased from the American Type Culture Collection (Manassas, VA). The *cdk4/hTERT*-immortalized normal human bronchial epithelial cell line, HBEC4 [16] was obtained from the Hamon Center Collection (University of Texas Southwestern Medical Center, Dallas, TX). AMJ2-C11 and LA-4 cells were cultured in Dulbecco’s Modified Eagle’s Medium supplemented with 100 U/ml penicillin, 0.1 U/ml streptomycin, 2.5x10^−4^ U/ml amphotericin B, 1 mM sodium pyruvate, Minimum Essential Medium (MEM) non-essential amino acids, and 10% Fetal bovine serum (FBS). HBEC4 cells were cultured in keratinocyte serum-free medium (Life Technologies, Gaithersburg, MD) supplemented with 50 ng/ml bovine pituitary extract and 5 ng/ml epidermal growth factor. Cells were grown under standard conditions in a humidified incubator at 37°C and 5% CO_2_.

### Animals

Wild-type BALB/c mice were purchased from SLC (Shizuoka, Japan). 8- to 12-week-old mice (weight: 18–25 g) matched for age and weight were used for the studies. Animals were maintained in a temperature (22-24°C), humidity (55 ± 5%), and light (12 hours light–dark cycle; lights on at 8:00) regulated room with access to food and water *ad libitum*. All procedures were performed in accordance with the Guidelines for Animal Experimentation of Nagoya University.

### Mouse model of ALI

Mice were randomly divided into 4 groups (each group: n = 3 ~ 5): control (Phosphate buffered saline (PBS))-treated, quercetin-treated, LPS-treated, and quercetin + LPS-treated. We made a small incision on the neck of mouse’s skin and exposed a trachea under sodium pentobarbital anesthesia. Then the mice were challenged intratracheally with either 50 μl PBS alone or 50 μl PBS of 1.25 μg LPS by stabbing the trachea with a microsyringe with a 22-gauge needle. In some experiments, mice were administered 50 μl of 0.1% propylene glycol (vehicle) or 10 μM quercetin in 0.1% propylene glycol intratracheally 6 hours before LPS challenge. The bronchoalveolar lavage fluid (BALF) and lungs were collected 24 hours after LPS administration in separate experiments. The severity of lung injury was assessed by wet lung-to-body weight ratio, pathological changes in lung tissues, and cellular profiles in BALF.

### Collection of BALF

BALF were collected as previously described [7]. Briefly, mice were exsanguinated by aortic perforation under pentobarbital anesthetization. The trachea was cannulated, and the lungs were lavaged six times with PBS (0.5 ml each time). Collected BALF were centrifuged at 1,200 rpm for 3 minutes, and the pelleted cells were then re-suspended in RPMI-1640 medium supplemented with 100 U/ml penicillin, 0.1 U/ml streptomycin, 2.5x10^−4^ U/ml amphotericin B, 1 mM sodium pyruvate, MEM non-essential amino acids, 50 μM 2-mercaptoethanol, and 10% FBS.

### Leukocyte counts in BALF

The number of leukocytes was enumerated with a hemocytometer. For differential counts, smears of BALF cells from each mouse were prepared with centrifugation using Cytofuge2 (StatSpin, Norwood, MA) at 1,000 revolution per minute for 2 minutes and then stained with May-Grünwald and Giemsa solutions.

### Wet lung-to-body weight ratio

The lungs were removed from the thoracic cavity and cleared of extraneous tissue. Each lung was weighed, and the wet lung-to-body weight ratio was then calculated to assess lung inflammation.

### Histological study

For histological examination, paraffin sections (6 μm thick) were stained with hematoxylin and eosin (H&E). For immunohistochemical examination, 3-μm sections were treated with 0.3% hydrogen peroxide, and then treated with 10% goat serum (Nichirei Biosciences, Tokyo, Japan) prior to incubating with a primary antibody against HO-1 (Enzo, Lausen, Switzerland) followed by the secondary antibody, Simple Stain Mouse MAX-PO (R) (Nichirei Biosciences). The slides were then visualized with DAB chromogen (Vector Laboratories, Burlingame, CA). The sections were counterstained with hematoxylin.

### Cell activation

As for HO-1 mRNA and protein expression in AMJ2-C11, LA-4, and HBEC4 cells, cells were cultured with various concentrations (0, 5, 10, 20 μM) of quercetin for 4 or 8 hours, respectively. As for the cytokine expression and production in AMJ2-C11 cells or BALF cells, cells were cultured with quercetin (AMJ-2C11 cells; 20 μM, BALF cells; 10 μM) for 1 hour and then stimulated with LPS (5 μg/ml) for 2 hours or 18 hours, respectively. In some experiments, AMJ-2C11 cells were treated with SnPP (20 μM) for 30 minutes before quercetin treatment. As for the MMPs activity and cytokine production in activated BALF cells in advance, BALF were collected 24 hours after an intratracheal LPS challenge and cultured with quercetin or vehicle for 1 hour. After the medium was changed, BALF cells were cultured with vehicle or quercetin for another 18 hours and the supernatant was collected.

### Western blotting

Western blotting was performed as described previously [14]. For analysis of HO-1 *in vivo*, lungs were collected 6 hours after intratracheal administration of vehicle or quercetin. For analysis of HO-1 *in vitro*, AMJ2-C11, LA-4, and HBEC4 cells were cultured with various concentrations (0, 5, 10, 20 μM) of quercetin for 8 hours.

### Reverse transcription (RT) - polymerase chain reaction (PCR), quantitative real-time PCR

Total ribonucleic acid (RNA) was isolated using ISOGEN II (Nippon Gene, Toyama, Japan) and reverse-transcribed to cDNA using PrimeScript RT MasterMix (Takara Bio, Shiga, Japan). Quantitative real-time PCR was performed on a Thermal Cycler Dice Real Time System II (TaKaRa Bio). Primers and probes for *Hmox1*, *Tnfa*, *Il1b*, *Il6*, *Gapdh, HMOX1,* and *GAPDH* were obtained from Nippon EGT (Toyama, Japan). Transcripts of *Gapdh* or *GAPDH*, as a house-keeping gene, were quantified as endogenous reference RNA to normalize each sample. Relative quantities of expression were estimated by the standard curve method. The results were normalized as relative expression, in which the average value of *Hmox1*, *Tnfa*, *Il1b*, *Il6*, or *HMOX1* was divided by the average value of *Gapdh* or *GAPDH*. The ratio was calculated by dividing the normalized values of stimulated cells by the values in control cells.

### Enzyme-linked immunosorbent assay (ELISA)

The cytokine production of TNF-α, IL-1β, and IL-6 in cell culture supernatants was quantified using a murine ELISA development kit (PEPROTECH, Rocky Hill, NJ) according to the manufacturer’s recommendations.

### Gelatin zymography

Gelatin zymography to determine matrix metalloproteinase (MMPs) activity was performed as previously described with some modifications [7]. The molecular weights of the gelatinolytic bands were estimated using Precision Plus Protein marker (BIO-RAD, Carlsbad, CA). The intensities of MMP-9 bands were estimated using Scion Image (Scion, Fredrick, MD).

### Statistical analysis

Statistical comparisons among the groups were assessed by one-way analysis of variance (ANOVA). When F ratios were significant (*p* < 0.05), Tukey-Kramer’s post-hoc test (between group comparison) were performed, and *p* < 0.05 was considered a statistically significant. Statistical analysis was performed with StatView (Abacus Concept Inc., Gloucestershire, UK).

## Results

### Lung protective effects of quercetin on LPS-induced ALI in mice

**Figure 1:**
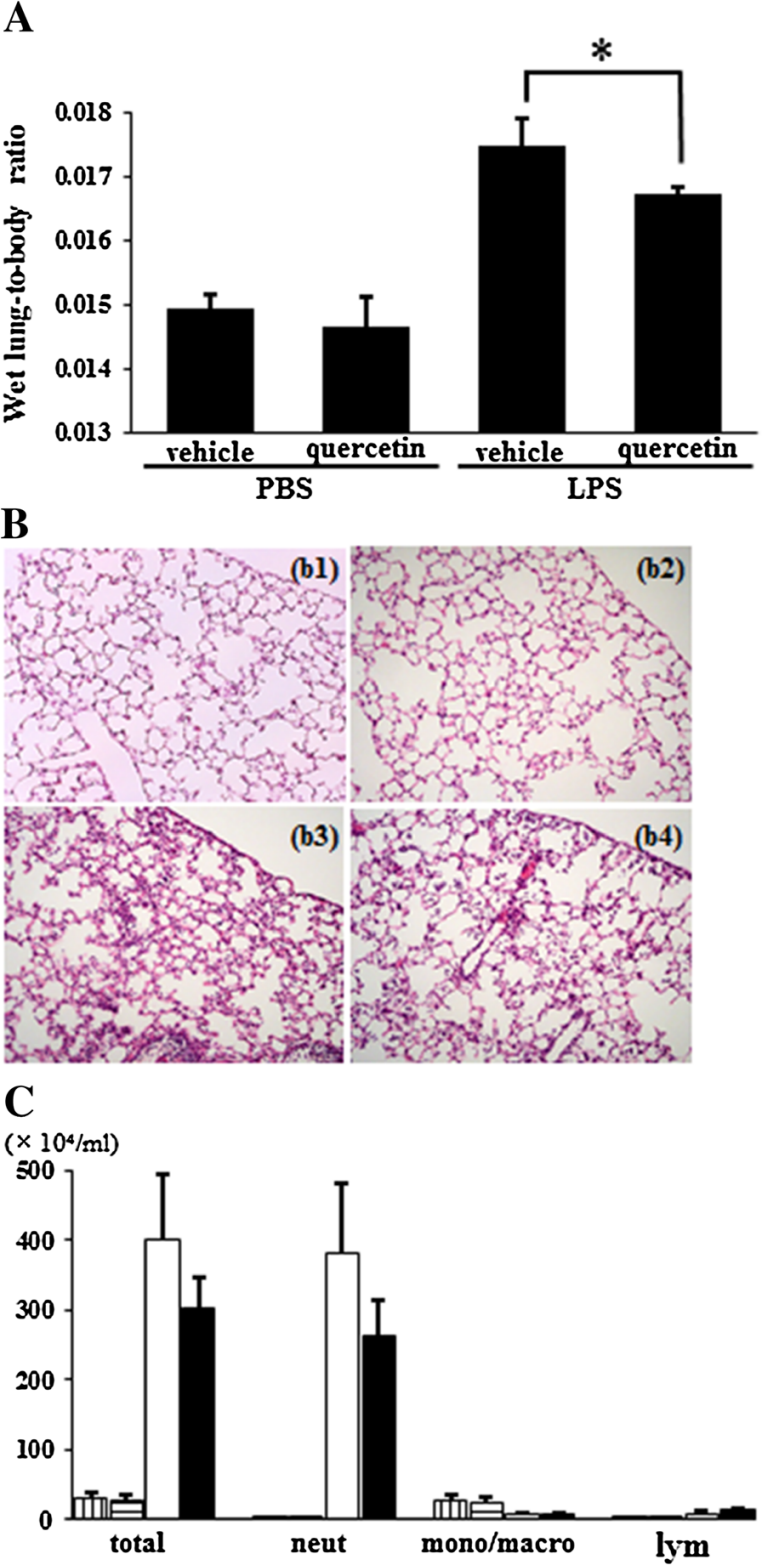
**The effects of quercetin on LPS-induced ALI.** Mice were challenged with LPS or PBS 6 hours after intratracheal administration of quercetin or vehicle. After 24 hours, the lung tissue and BALF were collected. **(A)** The wet lung-to-body weight ratio. **(B)** Histological images of lung tissues. (b1) vehicle-PBS; (b2) quercetin-PBS; (b3) vehicle-LPS; (b4) quercetin-LPS. *Original magnification*: ×100. Each photograph represents three independent experiments. **(C)** Inflammatory cell numbers in BALF. Total, total cell count; Neut, neutrophils; Mono/Macro, monocytes/macrophages; Lym, lymphocytes. *Vertically striped bars*, vehicle-PBS; *horizontally striped bars*, quercetin-PBS, *open bars*; vehicle-LPS, *filled bars*; quercetin-LPS. Results are shown as the mean ± SD (*p < 0.05) of three independent experiments.

We examined the effects of quercetin on LPS-induced ALI in mice. Mice were intratracheally challenged with LPS in the absence and presence of quercetin pretreatment. The wet lung-to-body weight ratio in LPS-challenged mice was significantly increased compared to control mice. The wet lung-to-body ratio was significantly decreased by quercetin pretreatment in mice challenged with LPS (Figure 1A). Lung histological sections of LPS-challenged mice showed alveolar wall thickening caused by edema, and marked and diffuse interstitial infiltration with neutrophils and monocytes/macrophages (Figure 1B, panel b3). Numbers of total cells and neutrophils in the BALF were increased in LPS-challenged mice (Figure 1C). Quercetin pretreatment reduced alveolar wall thickening and interstitial infiltration (Figure 1B, panel b4), and the number of total cells and neutrophils showed a decreasing tendency (Figure 1C); however, due to inter-animal variability, this was not statistically significant. These results indicated that quercetin pretreatment attenuated LPS-induced ALI in mice.

### HO-1 expression induced by intratracheal administration of quercetin in mouse lung

**Figure 2:**
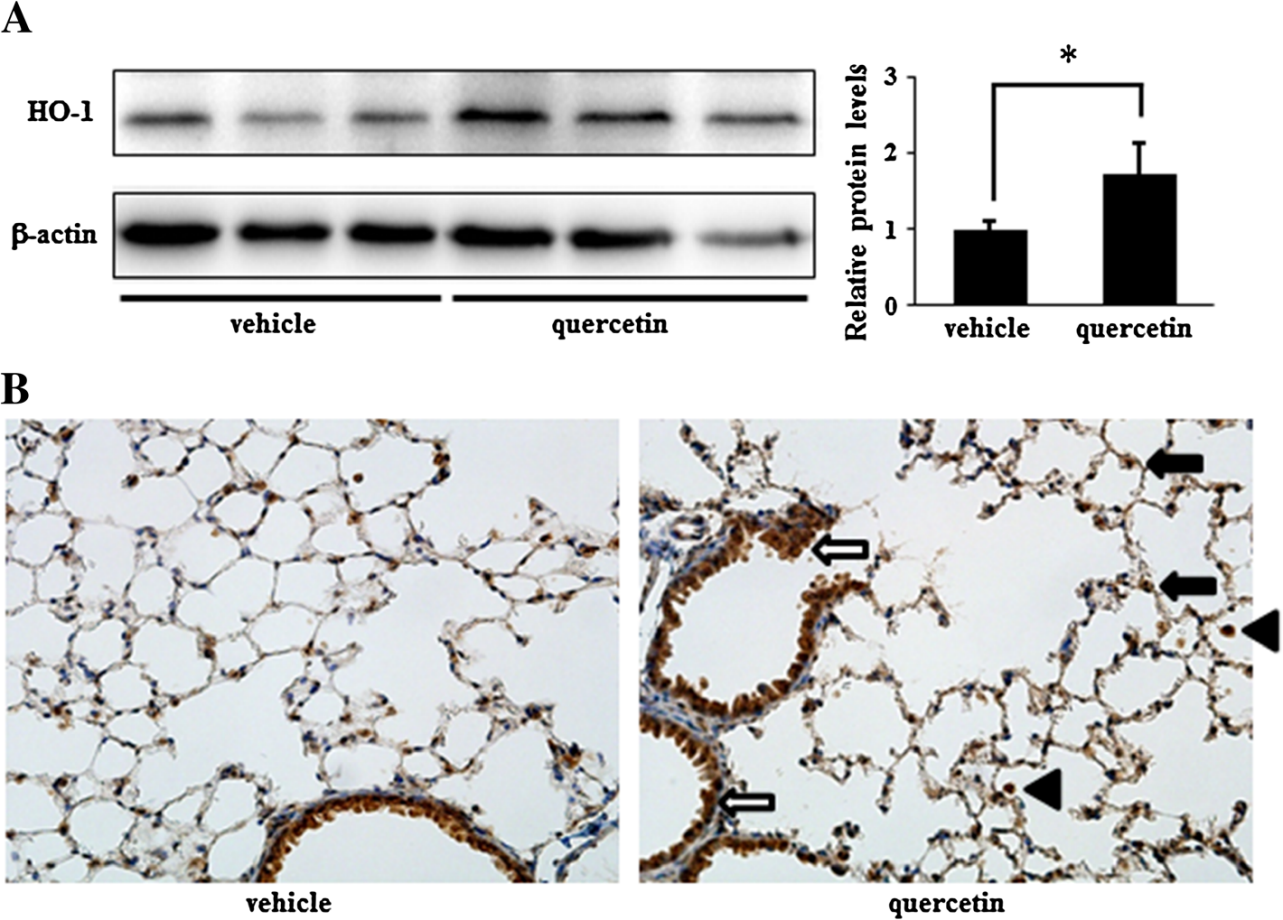
**The effects of intratracheal administration of quercetin on HO-1 expression in mouse lung.** Mice were sacrificed 6 hours after intratracheal administration of quercetin or vehicle. **(A)** Determination of HO-1 expression by western blotting. Results are shown as the mean ± SD (*p < 0.05) of three independent experiments. **(B)** Determination of HO-1 expression by immunohistrochemistry. *Arrowheads*, AMs; *black arrows*, alveolar epithelial cells; *open arrows*, bronchial epithelial cells. *Original magnification*: ×200. Each image is representative of three independent experiments.

We previously reported that quercetin exhibited cytoprotective effects via HO-1 induction [11,13]. Therefore, we next examined the expression of HO-1 in mouse lung 6 hours after intratracheal administration of quercetin using western blotting and immunohistochemistry. Figure 2A shows that the expression of HO-1 was significantly increased in lungs after administration of quercetin. In addition, increased expression of HO-1 was observed in AMs, alveolar and bronchial epithelial cells (Figure 2B). These results indicated that intratracheal administration of quercetin induced HO-1 in the mouse lung, especially in AMs, alveolar and bronchial epithelial cells.

### HO-1 expression induced by quercetin in AMs, alveolar and bronchial epithelial cells

**Figure 3:**
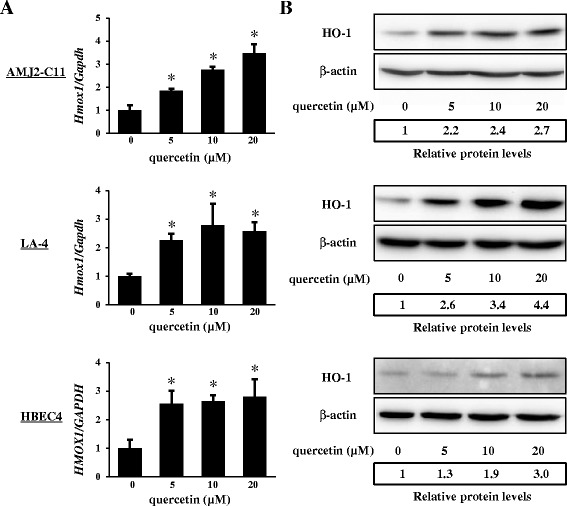
**The effects of quercetin on HO-1 expression in AMJ2-C11, LA-4, and HBEC4 cells.** AMJ2-C11, LA-4, and HBEC4 cells were cultured with various concentrations (0, 5, 10, 20 μM) of quercetin for 4 hours to detect HO-1 mRNA expression determined by quantitative real-time PCR **(A)**, and for 8 hours to detect HO-1 protein expression determined by western blotting **(B)**, respectively. Results are shown as the mean ± SD of three independent experiments. *Asterisks*, significantly different from the date at concentration zero of quercetin treated (*p* < 0.05).

We examined the induction of HO-1 mRNA and protein expression in AMs, alveolar and bronchial epithelial cells, using the mouse AM cell line AMJ2-C11, the mouse alveolar epithelial cell line LA-4, and the human bronchial epithelial cell line HBEC4, respectively. HO-1 expression, both at the mRNA level and at the protein level, was dose-dependently induced by quercetin in AMJ2-C11 cells. In AMJ2-C11 cells, at the highest quercetin concentration, the expression was increased up to 3.5-fold for mRNA and 2.7-fold for protein compared to control (Figure 3). Increased HO-1 expression by quercetin was also observed in LA-4 and HBEC4 cells. Consistent with results shown in Figure 2, these results suggested that HO-1 expression was significantly upregulated in lung tissue cells by quercetin.

### Suppressive effects of quercetin-induced HO-1 on LPS-induced pro-inflammatory cytokine production in AMJ2-C11 cells

**Figure 4:**
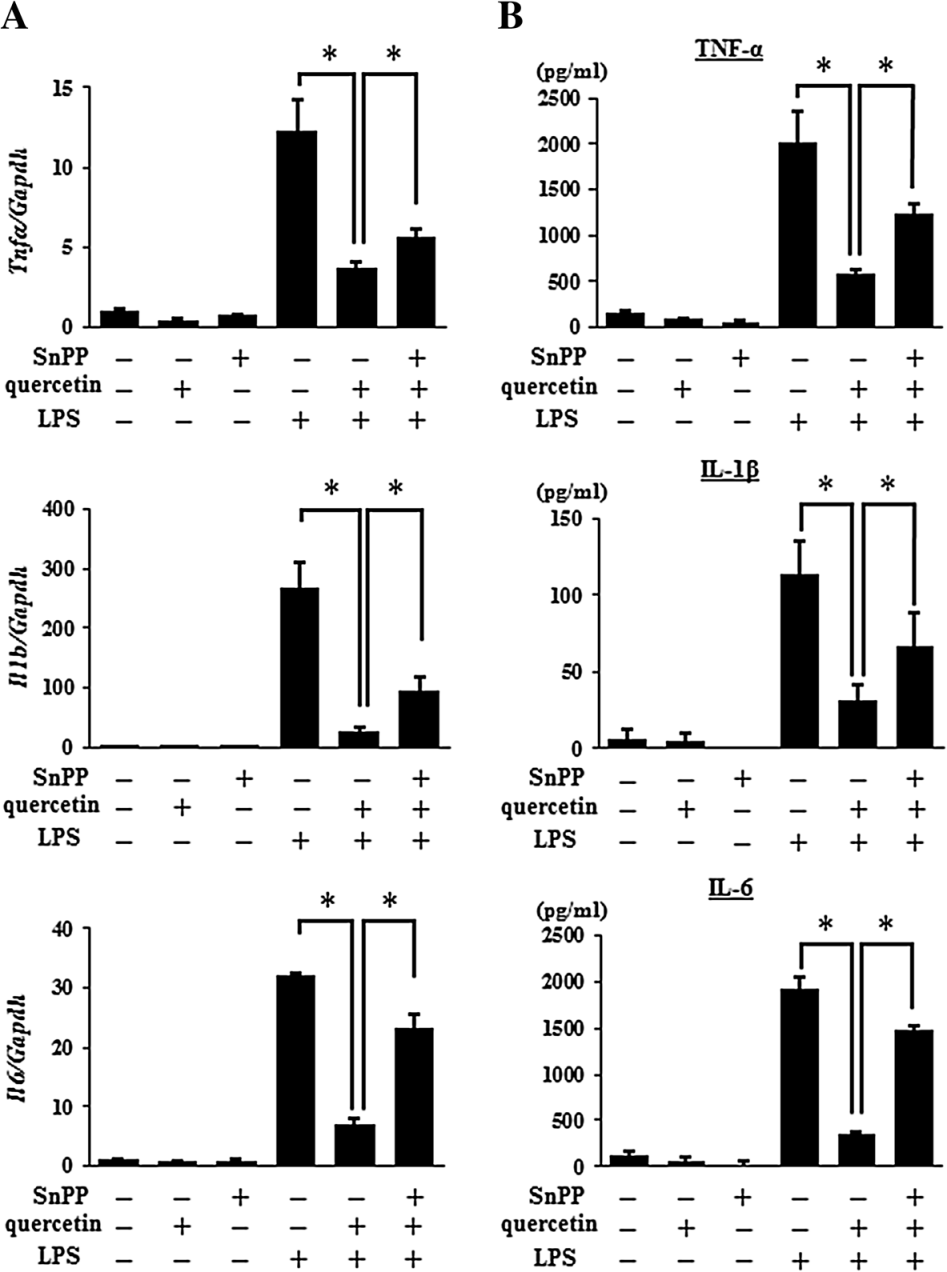
**The involvement of HO-1 in the suppressive effects of quercetin on LPS-induced pro-inflammatory cytokine production.** AMJ2-C11 cells were cultured with quercetin (20 μM) for 1 hour after treatment of SnPP (20 μM) for 30 minutes, and then stimulated with LPS (5 μg/ml) for 2 hours to detect the mRNA expression of TNF-α, IL-1β, and IL-6 determined by quantitative real-time PCR **(A)**, and for 18 hours to detect the production of TNF-α, IL-1β, and IL-6 determined by ELISA **(B)**, respectively. Results are shown as the mean ± SD (*p < 0.05) of three independent experiments.

From the results so far, we hypothesize that the induction of HO-1 could be associated with the biological potency of quercetin as an inhibitor of pro-inflammatory cytokines released by activated AMs and epithelial cells. AMs in the lung are known to be a critical modulator of inflammatory responses through the production of various pro-inflammatory cytokines [5,7]. To investigate the role of HO-1 on quercetin-induced inhibition of AMs activated by LPS, AMJ2-C11 cells were exposed to SnPP, an HO-1 inhibitor, before quercetin treatment and then stimulated with LPS. As shown in Figure 4, a marked suppression in the mRNA expression and production of TNF-α, IL-1β, and IL-6 induced by LPS was observed in quercetin-cultured AMJ2-C11 cells. The cells exposed to SnPP inhibited the quercetin-induced suppression of TNF-α, IL-1β, and IL-6 production. These results suggested that HO-1 was involved in the suppressive effects of quercetin on the production of LPS-induced pro-inflammatory cytokines in AMJ2-C11 cells.

### Suppressive effects of quercetin on LPS-induced pro-inflammatory cytokine production in BALF cells

**Figure 5:**
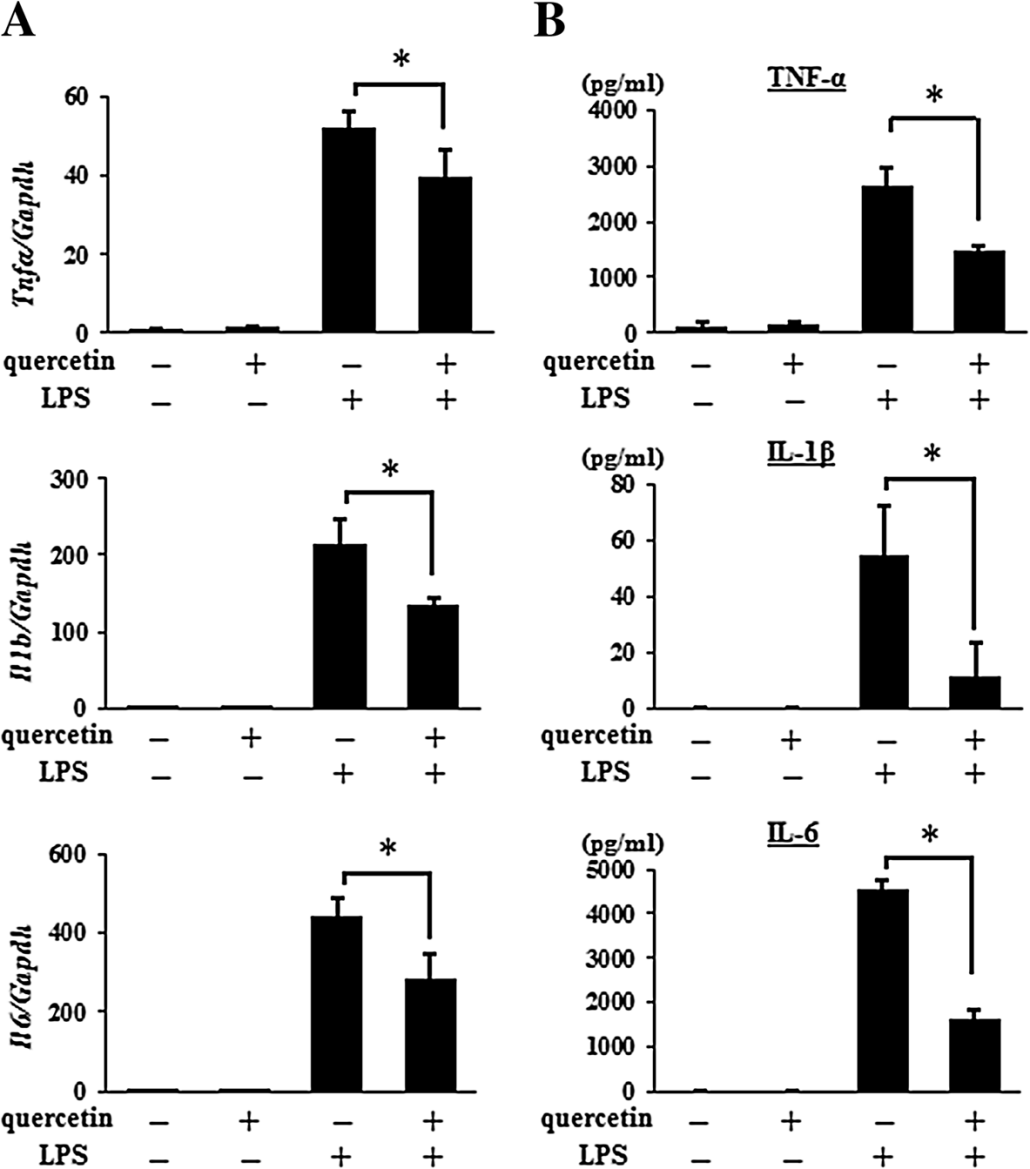
**The effects of quercetin on LPS-induced pro-inflammatory cytokine production in BALF cells** ***ex vivo*** **.** BALF cells cultured with quercetin (10 μM) or vehicle for 1 hour were stimulated with LPS (5 μg/ml) 2 hours to detect the mRNA expression of TNF-α, IL-1β, and IL-6 determined by quantitative real-time PCR **(A)**, and for 18 hours to detect the production of TNF-α, IL-1β, and IL-6 determined by ELISA **(B)**, respectively. Results are shown as the mean ± SD (*p < 0.05) of three independent experiments.

To further determine the suppressive effects of quercetin *ex vivo*, BALF cells were isolated from mice and stimulated with LPS after treatment with quercetin. Consistent with the results shown in Figure 4, the increase in mRNA expression and production of TNF-α, IL-1β, and IL-6 in BALF cells after stimulation with LPS was significantly suppressed by quercetin pretreatment (Figure 5). These results suggested that quercetin had cytoprotective effects *ex vivo* against the activation of AMs induced by LPS.

### Suppression of MMP-9 activity and pro-inflammatory cytokine production in LPS- induced ALI by quercetin

**Figure 6:**
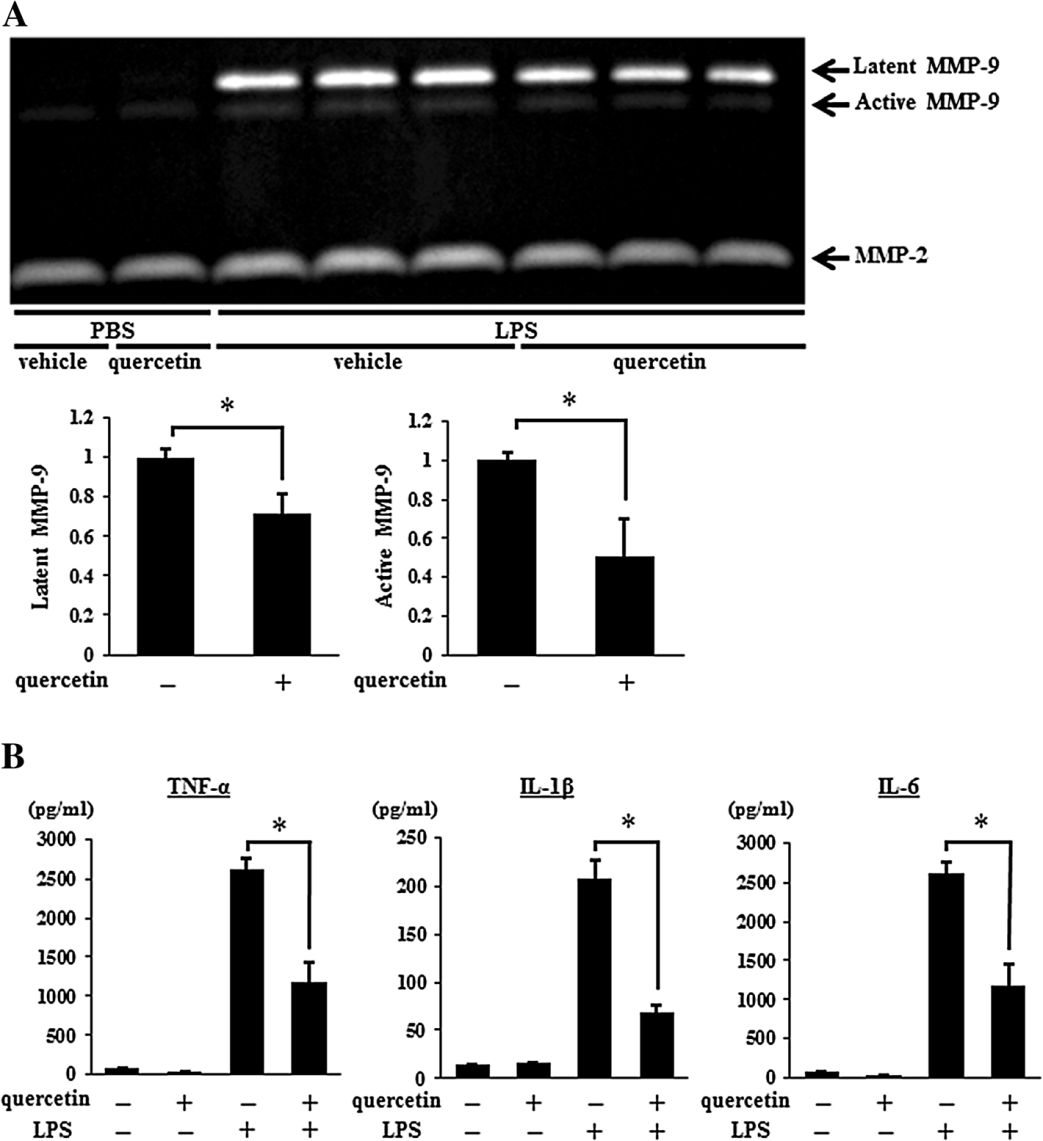
**The effects of quercetin on MMP-9 activity and pro-inflammatory cytokine production in LPS-induced ALI.** BALF were collected 24 hours after an intratracheal LPS challenge and cultured with quercetin or vehicle for 1 hour. After the medium was changed, BALF cells were cultured with vehicle or quercetin for another 18 hours and the supernatant was collected. **(A)** MMP-9 activity in the supernatant was determined by zymography. Latent (92 kDa) and active (88 kDa) MMP-9 and latent (72 kDa) MMP-2 activities are observed as clear zones of lysis against a dark background. MMP-9 activities were quantified by measuring the band intensities. **(B)** The production of TNF-α, IL-1β, and IL-6 in the supernatant was determined by ELISA. Results are shown as the mean ± SD (*p < 0.05) of three independent experiments.

To examine the effects of quercetin on BALF cells activated by LPS in advance, BALF were collected 24 hours after an intratracheal challenge with LPS and then treated with quercetin. As shown in Figure 6A, quercetin decreased both latent and active MMP-9 activities in BALF cells in LPS-treated mice, and the degree of suppression tended to be larger in active MMP-9 activities than in latent ones, but not statistically significant. We next examined the production of TNF-α, IL-1β, and IL-6 in the supernatant of BALF cells in LPS-treated mice. Consistent with the results in Figure 5, quercetin significantly reduced TNF-α, IL-1β, and IL-6 levels (Figure 6B). These results indicated that quercetin reduced the inflammatory responses in cells activated by LPS in advance *in vivo*.

## Discussion

In the current study, we first demonstrated that the intratracheal administration of quercetin attenuated LPS-induced ALI in mice and that quercetin suppressed LPS-induced pro-inflammatory cytokine production via an HO-1-dependent pathway. Furthermore, quercetin decreased the activity of MMP-9 and the production of pro-inflammatory cytokines in BALF cells activated by LPS in advance.

Intragastric administration of quercetin has been demonstrated to show preventive effects in LPS-induced sepsis in mice [17]. Although the daily dietary intake of quercetin has been estimated as 5–40 mg/day in humans [18], total quercetin derived from the diet was reported to be in the nanomolar range (<100 nM) in plasma as a result of its poor absorption and metabolism [19]. Furthermore, daily supplementation with 1 g of quercetin for 4 weeks increased plasma concentrations only up to 1.5 μM [20], which could not provide adequate amounts of quercetin to produce anti-inflammatory and anti-oxidant effects [19]. In consideration of these reports on the low bioavailability and the inability of patients with serious diseases, such as ARDS, to take quercetin orally, we administered quercetin intratracheally to maintain quercetin at high local concentrations.

We demonstrated that intratracheal prophylactic quercetin treatment suppressed the LPS-induced increase in the wet lung-to-body weight ratio. The wet lung-to-body weight ratio, an index of pulmonary edema, is correlated with the severity of lung injury, as previously reported [21]. Pulmonary edema, which is a major feature of ARDS, is associated with the malfunction of two lung cellular barriers: epithelial and endothelial cells [22]. Quercetin has been shown to improve the barrier function of both epithelial and endothelial cell [23,24]. Considering our results and previous reports together, the improvement of barrier function might be involved in the mechanism of suppression by quercetin on LPS-induced inflammation. Moreover, pro-inflammatory cytokines such as TNF-α, IL-1β, and IL-6 were reported to be increased in patients with ARDS, and play an important role in the initiation and propagation of the inflammatory cascade in ARDS [5,25]. Suppression of pro-inflammatory cytokine production by quercetin pretreatment may also have contributed to the attenuation of LPS-induced ALI in mice.

Lung protection as a consequence of HO-1 induction has been demonstrated in a number of lung injury models *in vitro* and *in vivo* [26]. HO-1 is an essential enzyme that catalyzes the degradation of heme to ferrous iron, CO, and biliverdin, which is subsequently converted to bilirubin [15]. The protective effects of HO-1 against inflammation are considered not only to decrease harmful heme, but also to produce the metabolites CO and bilirubin, which have the cytoprotective effects [27,28]. Importantly, quercetin induced the significant upregulation of HO-1 expression [13,14]. In fact, in this study quercetin induced HO-1 in AMs, alveolar and bronchial epithelial cells in mouse lung. Our results are consistent with previous reports that HO-1 expression was detected in AMs and epithelial cells in lungs with LPS stimulation or oxidative stress [29,30]. Furthermore, we confirmed the induction of HO-1 expression in these types of cells using cell lines. AMs and lung epithelial cells may produce pro-inflammatory cytokines locally in the lung in ARDS [5], so it makes sense that HO-1, which has anti-inflammatory effects, is expressed on these cells.

We investigated the activities of MMPs other than on the secretion of cytokines. MMP-9 is mainly produced by inflammatory cells, such as neutrophils and macrophages, and activation mechanisms of MMP-9 are involved in other MMPs, such as MMP-3, and neutrophil elastase [31,32]. The levels of MMP-9, which is a major factor in neutrophil migration across basement membranes [33], have been reported to be increased in patients with ARDS [34]. We observed that quercetin decreased the activity of MMP-9, the inhibition of which could mitigate the inflammatory response in lungs by suppressing the production of MMP-3 and neutrophil elastase [35,36]. In fact, mice lacking MMP-9 and the inhibition of MMP-9 have shown less lung injury [37,38]. Taken together, it is suggested that quercetin could suppress LPS-induced inflammation and have beneficial effects on lungs not only as a prophylactic treatment but also a supportive therapeutic drug.

## Conclusions

The present study demonstrated that the prophylactic effects of quercetin exhibited on LPS-induced lung inflammation, and that the HO-1-dependent pathway mediated these protective effects. Our findings indicate that intratracheal administration is a desirable method to effectively exert the cytoprotective effects of quercetin. Intratracheal administration of quercetin will lead to new supportive strategies for cytoprotection in serious conditions in the lung.

## Abbreviations

ALI: Acute lung injury
ARDS: Acute respiratory distress syndrome
HO: Heme oxygenase
LPS: Lipopolysaccharide
MMP: Matrix metalloproteinase
TNF: Tumor necrosis factor
IL: Interleukin
BALF: Bronchoalveolar lavage fluid
AM: Alveolar macrophage
CO: Carbon monoxide
SnPP: Tin protoporphyrin IX
MEM: Minimum essential medium
FBS: Fetal bovine serum
PBS: Phosphate buffered saline
H&E: Hematoxylin and eosin
SDS: Sodium dodecyl sulfate
PAGE: Polyacrylamide gel electrophoresis
RT: Reverse transcription
PCR: Polymerase chain reaction
RNA: Ribonucleic acid
ELISA: Enzyme-linked immunosorbent assay
SD: Standard deviation
